# Twisted magnon beams carrying orbital angular momentum

**DOI:** 10.1038/s41467-019-10008-3

**Published:** 2019-05-07

**Authors:** Chenglong Jia, Decheng Ma, Alexander F. Schäffer, Jamal Berakdar

**Affiliations:** 1 Key Laboratory for Magnetism and Magnetic Materials of the Ministry of Education and Institute of Theoretical Physics, Lanzhou University, 73000 Lanzhou, China; 2 Institut für Physik, Martin-Luther-Universität Halle-Wittenberg, 06099 Halle (Saale), Germany

**Keywords:** Nanoscale devices, Spintronics

## Abstract

Low-energy eigenmode excitations of ferromagnets are spin waves or magnons that can be triggered and guided in magnonic circuits without Ohmic losses and hence are attractive for communicating and processing information. Here we present new types of spin waves that carry a definite and electrically controllable orbital angular momentum (OAM) constituting twisted magnon beams. We show how twisted beams emerge in magnonic waveguides and how to topologically quantify and steer them. A key finding is that the topological charge associated with OAM of a particular beam is tunable externally and protected against magnetic damping. Coupling to an applied electric field via the Aharanov-Casher effect allows for varying the topological charge. This renders possible OAM-based robust, low-energy consuming multiplex magnonic computing, analogously to using photonic OAM in optical communications, and high OAM-based entanglement studies, but here at shorter wavelengths, lower energy consumption, and ready integration in magnonic circuits.

## Abstract (web summary)

The magnonic currents promise the opportunity for the energy efficient information processing and communication technologies. Here the authors theoretically show the propagating spin waves that carry orbital angular momentum can be electrically tunable and protected against damping in a cylindrical magnetic insulator waveguide.

## Introduction

**Fig. 1:**
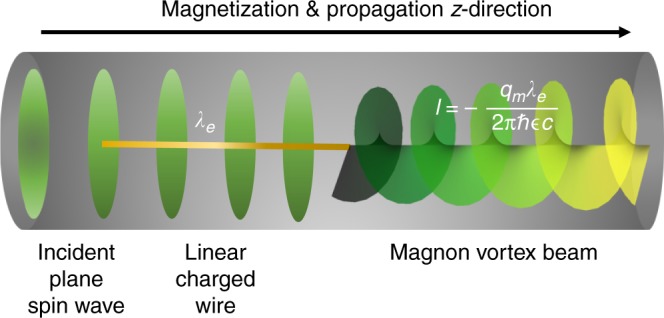
Proposal for generating a twisted magnon beam exploiting the Aharanov–Casher effect. Incident spin waves (impinging from left) that propagate in a cylindrical magnetic insulator waveguide traverse a region with a linear charge density *λ*_e_ (marked yellow). As demonstrated here, a magnonic twisted beam emerges to the right and can be quantified by the topological charge $\ell \propto \lambda _{\mathrm{e}}$, which is associated with a monopole-like vector potential, ${\mathbf{A}}_{\mathrm{e}}({\mathbf{r}})\sim \frac{{\lambda _{\mathrm{e}}}}{r}\widehat {\mathbf{e}}_\phi$ that is intimately related to the Dirac phase for magnons

Recently, the spatial structuring of photonic, electronic, or neutron beams has been demonstrated^1–5^ enabling to encode additional information in the beam spatial distribution. For instance, a photonic beam can be spatially modulated to have a helical or twisted wavefront embodying a well-defined, internal, meaning origin-independent, orbital angular momentum. The external orbital angular momentum (OAM) is origin dependent and is obtained from the photonic angular momentum density **L**_*γ*_ = **r**_*γ*_ × **P**_*γ*_, where **r**_*γ*_ is measured with respect to the beam center and **P**_*γ*_ is the linear momentum density^6^. Similar arguments apply to matter fields^3–5^. The intense research on this topic is fueled not only by fundamental interest but also by the new possibilities and functionalities accomplished by structuring the spatial distributions of the waves. For instance, OAM can take very large values that can be exploited for multiplex communications and quantum information based on entangled photons with large OAM^1–5,7–9^. Here we uncover the existence of twisted magnon beams, which are low-energy spin waves that carry a well-defined OAM. We demonstrate how they are triggered in a magnonic waveguide (such as in Fig. 1), and how to quantify their topological nature by an associated topological charge, which is found to be related to the OAM of a particular twisted beam. It is shown that this topological charge is electrically tunable and protected against damping. Hence, twisted magnonic beams are ideal candidates for robust, low-energy cost multiplex magnonic computing using the versatile toolbox for material and spin waves engineering^10–17^. In addition, we find an OAM-dependent magnonic Hall effect that depends on the value of OAM and, hence its value can be steered electrically. We also inspect the nature of thermal magnetic fluctuations around the equilibrium-ordered phase in a magnetic sample supporting twisted modes and find an anisotropic dependence of the correlation length.

## Results

### Analytical model for twisted magnon beams

What are the requirements for the emergence of OAM-carrying waves in magnetic systems and what is their relevance? In magnetically ordered systems key low-energy carriers of information are spin waves or magnons, which are the quanta of low-energy magnetic excitations. Identifying twisted magnonic beams that carry OAM would open a subfield in magnonics since OAM in principle can have an unbounded value. Thus, let us consider a generic ferromagnetic (FM) cylindrical waveguide modeled by localized magnetic moments at site *i* described by the magnetic moment operator **M**_*i*_ or the corresponding (dimensionless) spin operator **S**_*i*_ = −**M**_*i*_/(*γħ*) (*γ* is the gyromagnetic ratio). The (Heisenberg) Hamiltonian reads ${\cal{H}} = - \frac{1}{2}\mathop {\sum}\limits_{{ij}} {J_{{ij}}} {\mathbf{M}}_{i}\cdot {\mathbf{M}}_{j} - \frac{{K_{\mathrm{s}}}}{2}\mathop {\sum}\limits_{i} {(\widehat {\mathbf{e}}_{z}\cdot {\mathbf{M}}_{i})^2} - \mathop {\sum}\limits_{i} {\mathbf{B}} \cdot {\mathbf{M}}_{i}$, where *J*_*ij*_ > 0 is the exchange coupling between nearest-neighbor sites (*ij*), and *K*_s_ > 0 is a magnetic anisotropy contribution including the shape anisotropy (the demagnetizing factor tensor *D*_*ij*_ at any given direction of the cylindrical nanowire are approximately zero, except for *D*_*xx*_ = *D*_*y*y_ ≃ 1/2^18,19^). **B** = (0, 0, *B*_*z*_) is an external magnetic field along the waveguide (this direction is taken as the *z*-axis). For magnetically ordered systems longitudinal excitations, meaning changes in the expectation value of **M**_*i*_, caused for example by changes in *J*_*ij*_, are much higher in energy than transversal ones. The latter correspond to a precession of the unit vector field **m**_*i*_ = **M**_*i*_/|**M**_*i*_|, while |**M**_*i*_| = *M* = constant and their energy is typically set by *K*_s_, which is much smaller than *J*_*ij*_^20^. Hence, we inspect the transversal spin dynamics governed by the Heisenberg equation of motion ${\mathrm{d}}{\mathbf{m}}_{i}/{\mathrm{d}}t = - i/\hbar [{\mathbf{m}}_{i},{\cal{H}}]$. The dynamics of the magnetization unit vector field proceeds as (we introduce the effective field ${\mathbf{B}}_{\mathrm{s}}: = (K_{\mathrm{s}}M_{{iz}} + B_{z})\widehat {\mathbf{e}}_{z}$)

1 $$\frac{{{\mathrm{d}}{\mathbf{m}}_{i}}}{{{\mathrm{d}}t}} = \gamma \left[ {{\mathbf{m}}_{i} \times {\mathbf{B}}_{\mathrm{s}} - \mathop {\sum}\limits_{{j} \in {i}} M J_{{ij}}\left( {{\mathbf{m}}_{i} \times {\mathbf{m}}_{j}} \right)} \right].$$

We are interested in weak transversal fluctuations around the ordered magnetic phase (weak means slow variation in **m**_*i*_ within a lattice constant *a*). Furthermore, we will consider sufficiently large spins where quantum/thermal fluctuations are subsidiary and magnetic reversals are rare. Thus, it is useful to go over to a continuous magnetic texture vector field **m**_*i*_(*t*) → **m**(**r**_*i*_, *t*) and interpret **m**(**r**, *t*) as the local, time-dependent expectation value of the field operator **m**(**r**, *t*). The exchange contribution in Eq. (1) appears as the divergence of the spin current^21^, $\sum_{j} \gamma MJ_{{ij}}({\mathbf{m}}_{i} \times {\mathbf{m}}_{j}) \to \sum_{\mu \nu } {\partial _{\mu} } {\cal{J}}_{{m}_{\nu}}^{\mu}$. Here ${\cal{J}}_{{m}_{\nu} }^{\mu} = \gamma MJa^{2} \left[ {{\mathbf{m}} \times \partial _{\mu} {\mathbf{m}}} \right]_{\nu}$ stands for the *ν* polarized spin current along the *μ* spatial direction. With this Eq. (1) reads

2 $$\frac{{\partial {\mathbf{m}} ( {\mathbf{r}} ,{t})}}{\partial {t}} = \gamma {\mathbf{m}} ({\mathbf{r}},{t}) \times {\mathbf{B}}_{{\mathrm{s}}} - \sum_{\mu} {\partial _{\mu}} {{\cal{J}}}_{\mathbf{m}}^{\mu} ({\mathbf{r}},{t}).$$

The uniaxial symmetry of **B**_s_ dictates a conservation of the *z*-component of magnetization, meaning that $\partial _{t}{m}_{z} + {\nabla}\cdot {\cal{J}}_{{m}_{z}} = 0$. As discussed below, ${\cal{J}}_{{m}_{z}}$ is the only spin current with a non-vanishing time average. Within this general setting, we derive the equations governing the dynamics of small transverse deviations around the *z*-axis unit vector $\widehat{\mathbf{e}}_{z}$ by writing ${\mathbf{m}}({\mathbf{r}},{t}) \approx \widehat{\mathbf{e}}_{z} + \widetilde{\mathbf{m}}({\mathbf{r}},{t})$ with $\widetilde{\mathbf{m}} \bot\widehat{\mathbf{e}}_{z}$ and $|\widetilde {\mathbf{m}}| \ll 1$. With $\widetilde{\mathbf{m}} = (m_{x},m_{y},0)$ and inserting in Eq. (2) we infer the Schrödinger equation

3 $$i\hbar \partial _{t}\psi ({\mathbf{r}},t) = {\cal{H}}_{m}\psi ({\mathbf{r}},t),$$

where *ψ*(**r**, *t*) = *m*_*x*_(**r**, *t*) − *im*_*y*_(**r**, *t*) and

4 $${\cal{H}}_{m} = \frac{{{\mathbf{p}}^2}}{{2m_{J}^ \star }} + V(r),$$

where **p** = −*iħ***∇** is the momentum operator and $m_{J}^ \star = \hbar /(2\gamma JMa^2)$ is an effective mass. The wave function *ψ*(**r**, *t*) vanishes outside the waveguide and, otherwise, is subject to the potential *V*(**r**) = *−ħγ*(*K*_s_*M* + *B*_*z*_). In an extended cylindrical waveguide and in cylindrical coordinates **r** → (*r*, *ϕ*, *z*), Eq. (3) admits the non-diffractive Bessel solutions (see for instance ref. ^22^)

5 $$\psi ({\mathbf{r}},t) \propto J_\ell (k_ \bot r)\exp (i\ell \phi + ik_{z}z)\exp ( - i\omega t),$$

with $J_\ell (x)$ is the Bessel function of the first kind with order $\ell$. The total energy is

6 $$E({\mathbf{k}}) = \hbar \omega = \hbar ^2(k_{z}^{\mathrm{2}} + k_ \bot ^2)/2m_{J}^ \star + V.$$

Note that similar to optics, conventional (meaning $\ell = 0$ in Eq. (5)) Bessel modes^22,23^ also exist, but those do not carry any OAM. A striking consequence is that Bessel beams do not interact with electric fields, in contrast to twisted magnon beams and also do not allow for $\ell$-based operations. This fact is decisive when it comes to constructing OAM-sensitive magnonic circuits (similar to ref. ^11^) that are controlled by electric fields offering so new functionality, as discussed in ref. ^24^. A further important aspect is the difference between our propagating OAM modes and static/driven magnetization vortices^25–27^. The latter are localized topological excitations that are much higher in energy than a magnon wave (cf. Eq. (5)), which extends over the whole waveguide. The much lower energy set by *E*(**k**) is tunable by $J,m_{J}^{\star}$ and *V*. Both magnetization vortices and propagating, OAM-carrying magnons have well-defined topological features that can be quantified. To do that in our case, we consider the *z*-component of the magnon current ${{\cal{J}}}_{{m}_{z}}$, which is nothing but the probability current density coiling around the *z*-axis

7 $${{\cal{J}}}_{{{m}}_{{z}}} = \hbar \Re \left[ {\psi ({\bf{r}},t)\frac{{\mathbf{p}}}{{m_{{J}}^{\star} }}\psi ^{\star} ({\mathbf{r}},t)} \right] \propto \left( {\frac{\ell }{r}\widehat {\mathbf{e}}_\phi + k_{z}\widehat {{e}}_{z}} \right)\rho _{\ell} (r),$$

meaning that the spin waves spiral around the axis of the waveguide, similarly to the case of photon, electron, or neutron twisted beams^1–5^. In fact, the analogy runs deeper: let us introduce pseudo-electric and -magnetic fields as $\widetilde {\mathbf{E}} = {\mathbf{m}}_{x} + i{\mathbf{m}}_{y}$ and $\widetilde {\mathbf{B}} = i({\mathbf{m}}_{x} - i{\mathbf{m}}_{y}).$ Physically, $\widetilde {\mathbf{B}}$ and $\widetilde {\mathbf{E}}$ describe the (paraxial) spin wave modes rotating clockwise and anticlockwise, respectively. The magnon density is ${\cal{W}}_{m} = \frac{1}{2}\left( {|\widetilde {\mathbf{E}}|^2 + |\widetilde {\mathbf{B}}|^2} \right)$, and the *m*_*z*_ polarized magnon current density ${{\cal{J}}}_{{{m}}_{{z}}}$ can be related to the pseudo-Poynting-like vector^28^, ${\cal{P}}_\mu : = {\cal{J}}_{{m}_{z}}^\mu \propto \frac{1}{2}\left[ {\widetilde {\mathbf{E}}^ \star \times (\partial _\mu \widetilde {\mathbf{B}}) + \widetilde {\mathbf{B}}^ \star \times (\partial _\mu \widetilde {\mathbf{E}})} \right]$. It is straightforward to infer that ${\cal{P}}$ has the proper time and space symmetry of a canonical momentum. Noting that our twisted beam is localized within the waveguide, we can introduce the canonical OAM of the magnon twisted beam as ${\mathbf{L}} = {\mathbf{r}} \times {\cal{P}}$, which is extrinsic and dependent on the choice of the coordinate origin. However, the integral orbital angular momentum over the cross-section is indeed intrinsic, amounting to

8 $$\langle {{\cal{P}}}\rangle \propto \langle {\mathbf{k}}\rangle ,\quad \langle {\mathbf{L}} \rangle \propto \ell \langle {\mathbf{k}} \rangle {/} {k},$$

with ${\mathbf{k}} \simeq k_{z}\widehat {\mathbf{e}}_{z}$ being the mean wave vector of the spin wave. We have then $\langle {\mathbf{L}}\rangle \cdot \langle {\cal{P}}\rangle /{\cal{P}} = \ell$, in full analogy with paraxial optical twisted beams^28^. This $\ell$ can be identified with the topological charge associated with a particular twisted beam. Generally, our magnons are characterized by the integral momentum $\langle {\kern 1pt} {\cal{P}}{\kern 1pt} \rangle$ and the orbital angular moment 〈**L**〉 as two independent properties, meaning that in addition to the intrinsic spin angular moment *ħ* along the $\widehat {\mathbf{e}}_{z}$, the magnon in the cylindrical nanowire may carry a longitudinal and intrinsic orbital angular moment $L_{z}\sim \hbar \ell$ as well, which formally can take arbitrarily large values and can be utilized in magnonic and spintronics applications.

### Undamped topological charge of twisted beams

An important feature of twisted magnons is the robustness of the associated topological charge to magnetic damping. This fact is crucial when it comes to utilization of the OAM of twisted magnons as information carriers, analogously to OAM-based optical communications^7,8^. Generally, the (Gilbert) damping of magnetization procession^11,17^ is governed by a term of the form *α***m** × *∂***m**/*∂*_*t*_. In Eq. (3), this amounts to substituting *∂*_*t*_ → (1 + i*α*)*∂*_*t*_, which leads to the time-decaying magnon density $\rho _\ell (t) = \rho _\ell (t = t_0)\exp ( - 2\alpha \omega (t - t_0))$ when propagating from time *t*_0_ to *t*. The magnon OAM $\ell$ derives, however, by an averaging of 〈**L**〉 over the cross-section of the waveguide *S*, meaning $\langle {\mathbf{L}}\rangle = \langle {\mathbf{r}} \times {\mathbf{P}}\rangle _{S} = \hat e_{z}({\int} \ell \rho _\ell (t){\mathrm{d}}S)/({\int} {\rho _\ell } (t){\mathrm{d}}S) = \ell \hat e_{z}$. Thus, damping leads to a decaying magnon density (its photon analog is the light intensity), which is however independent of $\ell$ and hence of OAM. In other words, the OAM ($\ell$) is not affected by damping and is conserved during the magnetization evolution. Another point of view is that the damping term would dominate the interfacial spin-pumping current by ${\mathbf{m}} \times \partial {\mathbf{m}}/\partial t \propto \Im \left[ {\psi ({\mathbf{r}},t)\partial _t\psi ^ \star ({\mathbf{r}},t)} \right] = \omega \rho _\ell$, which clearly does not depend on the internal phase structure of the twist excitation (and hence on $\ell$). These conclusions are supported by the full-fledge numerical simulations presented below.

### Simulating an experimental realization

Having laid down the principle existence of twisted OAM and the robustness of the associated topological charge to perturbations that subsume into damping of the precessional dynamics, the experimental feasibility as well as the role of further magnetic interactions that emerge in a realistic setting, have to be clarified. Therefore, we resort to full-fledge numerical micromagnetic simulations (see Methods) for an experimentally realistic waveguide made of insulating yttrium iron garnet (YIG).

**Fig. 2:**
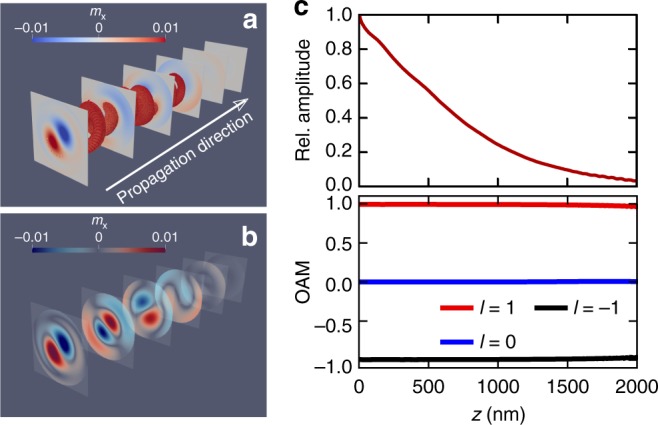
Spiraling spin wave propagating along a cylindrical micro-scale waveguide of the insulting magnet yttrium iron garnet. **a** Snapshot of the magnon beams after 2 ns for the *x*-component of the triggered magnetization. **b** Vortex configuration of the excitation modes along the waveguide. **c** The *z*-resolved amplitude and the orbital angular momentum of the spin waves. In **a**–**c**, the spin waves are excited locally at one end of the waveguide by a twisted radio frequency (rf) magnetic field having the peak amplitude *B*_max_ = 10 mT, and a frequency *ω*_*B*_ = 5 GHz. For the time evolution of *m*_*x*_ at the transversal slice *z* = 1 μm see Supplementary Movie 1

The cylindrical YIG waveguide has a diameter of 0.4 μm and a length of 2.0 μm. The saturation magnetization is *M*_s_ = 1.4 × 10^5^ A/m, the exchange stiffness constant is *A*_ex_ = 3 × 10^−12^ J/m, the uniaxial magnetocrystalline anisotropy along *z* is *K*_u_ = 5 × 10^3^ J/m^-3^, and the Gilbert damping *α* = 0.01^24^. The ground state equilibrium magnetization is oriented along the +*z*-axis, parallel to the main axis of the waveguide. To launch the spin waves, we excite locally at one end *z* = 0 with a twisted magnetic *B* field (see Methods) that is linearly polarized in the *x*-direction and having the amplitude *B*_max_ = 10 mT and the frequency *ω*_*B*_ = 5 GHz. Figure 2a shows a snapshot of the *x*-component of magnetization excitations propagating after 2 ns. More details of excitation mode are gained from slices along the tube in Fig. 2b, which demonstrates the generation of propagating twisted magnon beams. An animated version of *m*_*x*_ at the transversal slice *z* = 1 μm during the magnon propagation is provided as Supplementary Movie 1. The finite damping leads to a decaying magnon density along the YIG tube with the amplitude being normalized with respect to the maximum excitation at *z* = 0. Remarkably, when evaluating the OAM $\ell$ from the numerically calculated pseudo-Poynting vector, we indeed find that $\ell$ is independent of the magnon density and is conserved during the spin wave propagation along the tube (cf. Fig. 2c). These full numerical calculations endorse the above formal analysis as well as the experimental feasibility and relevance of twisted magnon beams.

### Aharonov–Casher effect and Landau levels

The simulations shown in Fig. 2 as well as our formal analysis evidence that the twisted magnon beams embody a non-vanishing *z*-polarized spin current density allowing for a coupling to an external electric field ${\cal{E}}$ through the Aharonov–Casher (AC) effect^29^. For magnon-based information transfer, this fact is crucial as it allows to control the flow of OAM-carrying magnons with electric means. In the presence of an electric field, the exchange interaction in the Heisenberg Hamiltonian becomes anisotropic according to $J_{ij}{\mathbf{M}}_{i}\cdot {\mathbf{M}}_{j} \to \frac{1}{2}J_{ij}\left( {M_{i}^{\mathrm{ + }}M_{j}^ - {\mathrm{e}}^{i\theta _{{ij}}} + M_{i}^ - M_{j}^ + {\mathrm{e}}^{ - i\theta _{{ij}}}} \right) + J_{{ij}}M_{i}^zM_{j}^z,$ where $\theta _{ij} = \frac{{g\mu _{\mathrm{B}}}}{{\hbar {c}^{2}}}{\int}_{{\mathbf{r}}_{i}}^{{\mathbf{r}}_{j}} {\mathrm{d}} {\mathbf{r}}\cdot ({{\cal{E}}} \times {\widehat{{\mathbf{e}}}_{z}})$ is the AC phase. The canonical momentum **p** in Eq. (4) has then the kinetic momentum form ${{\frak{p}}}_{\mathrm{e}} = {\mathbf{p}} - q_{\mathrm{m}}{\mathbf{A}}_{\mathrm{e}}$, where *q*_m_ = *gμ*_B_/*c* and the electric vector potential **A**_e_ is given by ${\mathbf{A}}_{\mathrm{e}}({\mathbf{r}}) = - {{\cal{E}}}({\mathbf{r}}) \times {{\widehat {{\mathbf{e}}}_{{z}}}}/c$. For the electric field ${\cal{E}}({\mathbf{r}}) = {\cal{E}}(x/2,y/2,0)$, one finds the symmetric twist vector potential ${\mathbf{A}}_{\mathrm{e}}({\mathbf{r}}) = \frac{{\cal{E}}}{c}( - y/2,x/2,0) = \frac{{{\cal{B}}r}}{2}\widehat {\mathbf{e}}_\phi .$ Our case is analogous to an electron system in uniform perpendicular magnetic field $\sigma |{\cal{B}}|\widehat {\mathbf{e}}_z$^30^, and $\sigma = {\mathrm{sign}}({\cal{E}})$ is set by the direction of the applied electric field ${\cal{E}}$. From the gauged Hamiltonian ${\cal{H}}_{\mathrm{m}} = {{\frak{p}}}_{\mathrm{e}}^2/2m_{J}^ \star$ follow the well-known quantized Landau states having the form of non-diffracting Laguerre–Gaussian (LG) modes^31–33^

9 $$\psi _{\ell ,{n}}^L(r,\phi ) \propto \left( {\frac{{\sqrt 2 r}}{{w_{\mathrm{e}}}}} \right)^{|\ell |}L_{n}^{|\ell |}\left( {\frac{{2r^2}}{{w_{\mathrm{e}}^{\mathrm{2}}}}} \right)\exp \left( { - \frac{{r^2}}{{w_{\mathrm{e}}^{\mathrm{2}}}}} \right){\mathrm{e}}^{i\ell \phi + ik_{z}z},$$

where $L_{n}^{|\ell |}$ are the associated Laguerre polynomials, and *n* = 0, 1, 2, … is the quantized radial number. The Landau energy levels read

10 $$E_{k}^L = \frac{{\hbar ^2k_{z}^{\mathrm{2}}}}{{2m_{J}^ \star }} - \hbar \Omega _{\mathrm{e}}\ell + \hbar |\Omega _{\mathrm{e}}|\left( {2n + |\ell | + 1} \right).$$

Here $w_{\mathrm{e}} = 2\sqrt {\hbar /q_{\mathrm{m}}|{\cal{B}}|}$ is a characteristic width that depends on the amplitude of the applied electric field, $\Omega _{\mathrm{e}} = q_{m}\sigma |{\cal{B}}|/2m_{J}^ \star$ is the Larmor frequency (*σ* = ±1 correspond to the clockwise or anticlockwise rotation, respectively). Thus, the electric field ${\cal{E}}$ confines the magnons to form paraxial LG beams with

11 $${{\cal{J}}}_{m_{{z}}}^L \propto \left[ {\frac{1}{r}\left( {\ell + \sigma \frac{{2r^2}}{{w_{\mathrm{e}}^{\mathrm{2}}}}} \right)\widehat {\mathbf{e}}_\phi + k_{z}\widehat {\mathbf{e}}_{z}} \right]\rho _{\ell ,{{n}}}^L(r,z).$$

The azimuthal component is directly related to the kinetic orbital angular momentum ${\cal{L}} = {\mathbf{r}} \times {\cal{P}}_{\mathrm{e}}$ as $\langle {\cal{L}}_{z}\rangle = - \hbar \langle i\partial _\phi + q_{\mathrm{m}}rA_{\mathrm{e}}\rangle$. Unlike the Bessel beams with uniform rotational direction described by the topological charge $\ell$, the direction of $\langle {\cal{L}}_{z}\rangle$ of the LG beams depends on $\ell$ and the twist vector potential **A**_e_ as well. Note, the $\langle {\cal{L}}_{z}\rangle$ radial structure depends strongly on *σ*, that is, the direction of the applied electric field in terms of the first radial maximum of the LG mode $r_{|\ell |} = w_{\mathrm{e}}\sqrt {|\ell |/2}$: For $\ell \sigma > 0$, we have a uniform, rotationally azimuthal current as usual. However, for $\ell \sigma < 0$ the currents in the region of $r < r_\ell$ and $r > r_\ell$ are counter-circulating and are dominated by the topological charge $\ell$ and the twist vector potential **A**_e_, respectively. For an estimate, we take: *JM*^2^ = 0.1 μeV, *a* = 10 Å, and ${\cal{E}} = 1$ V/nm, which give *w*_e_ ≈ 1.4 μm and *ħ*|Ω_e_| ≈ 0.33 μeV. Increasing the strength of the applied electric field, higher electric Larmor frequencies Ω_e_ are achievable but the electric Landau radius *w*_e_ is smaller.

### OAM-tunable magnon Hall effect

Having clarified the role of an external electric field, one may wonder how the twisted magnon beam is affected by magnetic perturbations. A hallmark of a charge wave-packet propagation in a magnetic field is the transverse deflection, that is, the Hall effect, which in its simplest variant is assigned to the Lorentz force. A similar effect for uncharged particles, such as photons, phonons, or magnons, may thus appear surprising. In our FM case, on the other hand, the Hall effect is also sensitive to the residual magnetization, a phenomenon termed the anomalous Hall effect. Indeed, the Hall effect for magnons has already been observed^34^. Is this effect OAM dependent and can we control it by tuning the OAM? A positive answer would be an important advance, since OAM can be controlled externally, as demonstrated in Fig. 2. To approach this issue, we note that LG modes have well-defined azimuthal and radial wavefront distributions (quantified by $\ell$ and *n*, respectively) and they form an orthogonal and complete basis in terms of which an arbitrary function can be represented. So let us consider a magnonic wave packet centered at (**p**_*c*_, **r**_*c*_) (similar to electronic wave packets^35,36^). From the above, we conclude that the trajectory spirals around the *z*-axis. Projecting the local coordinate frame along the curved trajectory, we end up with a noncommutative geometry and a covariant derivative in momentum space^37–40^: $D_{i} = \partial /\partial _{{p}_{i}} + {\cal{A}}_{i}^g({\mathbf{p}})$, where ${{\mathbf{\cal{A}}}}^g({\mathbf{p}}) = i\langle \psi _{\ell ,{\mathrm{n}}}^L|\partial /\partial _{\mathbf{p}}|\psi _{\ell ,{\mathrm{n}}}^L\rangle$ is the Berry connection. The corresponding Berry gauge field (curvature), ${\cal{B}}_{\mathrm{g}} = \partial/\partial _{\mathbf{p}} \times {\cal{A}}^{g}({\mathbf{p}})$ gives rise to a deflection of the wave packet center. Given that the *z*-axis is now locally directed along **p**, the vector $({\widehat {\mathbf{e}}}_{x} + i{\widehat {\mathbf{e}}}_{y})$ is orthogonal to **p** and moves on the unit sphere with **p**/*p* under variation of **p**, which results in a magnetic-monopole-type field^41,42^, ${{\cal{B}}}_{{\mathrm{g}}} = - \ell {\mathbf{p}}/p^{3}$ for the orbital motion of magnons (since $\exp (i\ell \phi) = ({\widehat {\mathbf{e}}}_{x} + i{\widehat {\mathbf{e}}}_{y})^{\ell}$). As a result, we infer the Hamiltonian equations of motion^35,39^

12 $$\frac{{\mathrm{d}}{r}_{c}}{{\mathrm{d}}{t}} = \frac{{\partial H}}{{\partial {{p}}_{{c}}}} - \hbar {\dot{{p}}}_{{c}} \times {{\cal{B}}}_{{\mathrm{g}}} = \frac{{{{p}}_{{c}}}}{{m_{{J}}^ \star }} + \hbar \ell {\dot{{p}}}_{{c}} \times \frac{{{{p}}_{{c}}}}{{p_{{c}}^3}},$$

13 $$\frac{{\mathrm{d}} {\mathbf{p}}_{c}}{{\mathrm{d}}{t}} = - \frac{\partial{H}}{\partial {\mathbf{r}}_{c}}= - \frac{\partial V(r)}{\partial {\mathbf{r}}_{c}} - \frac{q_m}{cm_{J}^{\star}}\frac{\partial}{\partial {\mathbf{r}}_{c}}\left[ {\mathbf{p}}_{c} \cdot ({{\cal{E}}} \times {\hat{{\mathbf{e}}}}_{z})\right].$$

The anomalous velocity term $\hbar \ell {\dot{\mathbf{p}}}_{c} \times {\mathbf{p}}_{c}/p^3$ is perpendicular to **p**_*c*_ causing a transversal motion of the wave packet. The corresponding transverse shift *δ***r**_*c*_ is cast as $\delta {\mathbf{r}}_{c} = \hbar \ell {\int}_{L} {({\mathbf{p}}_{c} \times {\mathrm{d}}{\mathbf{p}}_{c})} /p_{c}^{\mathrm{3}}$ and is determined by the geometry of the contour *L* in momentum space and $\ell$. This is in so far remarkable, as arbitrarily large values of $\ell$ are possible giving rise to a large deflection at small driving force.

**Fig. 3:**
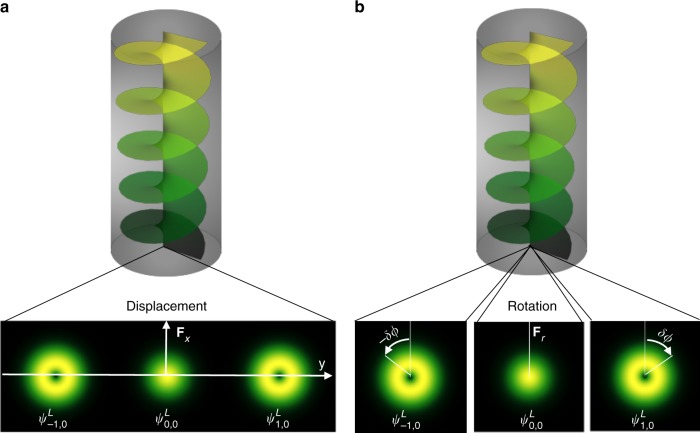
Demonstration of the orbital angular momentum (OAM)-dependent magnon Hall effect. **a** Transverse displacement by a driving force **F**_*x*_ along the *x*-direction and **b** uniform rotation by a *r*^2^-inhomogeneity

For concreteness, let us consider a weak spatially varying, perpendicular magnetic field *B*_*z*_, say along the *x*-direction^43,44^. The driving force is ${\mathbf{F}}_{x} = - \partial _{x}B_{z}({\mathbf{r}})\widehat {\mathbf{e}}_{x}$ and thus ${\dot{\mathbf{p}}}_{c} = {\mathbf{F}}_{x}$ and $\delta {\dot{\mathbf{r}}}_{c} = \hbar \ell {\mathbf{F}}_{x} \times {\mathbf{p}}_{c}/p_{c}^{\mathrm{3}}$. Assuming paraxial magnonic propagation, meaning ${\mathbf{p}}_{c} \simeq p_{z}\widehat {\mathbf{e}}_{z}$, the transverse deflection reads (an exact but cumbersome analytical expression is available) $\delta {\mathbf{r}}_{c} \simeq \delta {\mathbf{y}} \propto \frac{{\hbar \ell F_{x}z}}{{p_{c}^3}}\widehat {\mathbf{e}}_y.$ Different LG modes with different topological charges $\ell$ split at the waveguide end (cf. Fig. 3a). Similar to photon^41^ and electron^42^ twisted beams, we predict a magnonic OAM-dependent Hall effect, which is qualitatively different from the usual magnon Hall effect^10,34^.

For a cylindrically inhomogeneous waveguide, for instance due to inhomogeneity in the demagnetization field^18,19^, we may formally write the potential in Eq. (4) as $V(r) = \bar V + V_2r^2/2$ with $|V_2| \ll |\bar V|$ causing further $\delta {\mathbf{r}}_{c} = \delta {\boldsymbol{\phi }} \propto \frac{{\hbar \ell V_2z}}{{p_{c}^3}}\widehat {\mathbf{e}}_\phi .$ The displacement direction is along $\widehat {\mathbf{e}}_\phi$, meaning that LG modes are rotated uniformly clockwise or anticlockwise depending on $\ell$ (cf. Fig. 3b), a result in line with the optical Magnus effect^6,45^.

Another aspect arises when the waveguide is an FM metallic wire, in which case the electric field vanishes inside the wire. However, we may still have nontrivial magneto-electric effects. Starting from a general symmetrical but inhomogeneous electric potential *V*_e_(*r*), the energy shift due to the AC effect is given to the first order in the perturbation *V*_e_(*r*) by $\delta E \simeq \frac{{q_{m}}}{{m_{J}^ \star c}}\langle \frac{1}{r}\frac{{\partial V_{\mathrm{e}}}}{{\partial r}}\left[ {\widehat {\mathbf{e}}_{z}\cdot ({\mathbf{r}} \times {\mathbf{p}})} \right]\rangle = \frac{{q_{m}}}{{m_{J}^ \star c}}\langle \frac{{L_{z}}}{r}\frac{{\partial V_{\mathrm{e}}}}{{\partial r}}\rangle .$ For a step-function profile of the wire electric potential *V*_e_(*r*) = *U*Θ(*r* − *R*) (here Θ(*r*) is the Heaviside function and *R* is the radius of the metallic wire), we find *∂V*_e_/*∂r* = *Uδ*(*r* − *R*). No electric field is present in the nanowire except for the surface. Nonetheless, the twisted magnon beam acquires an additional phase upon traveling a distance *z* along the nanowire $\varphi (\ell ,z) = U\rho _\ell (R)\frac{{q_{m}}}{{\hbar c}}\frac{\ell }{{k_{z}}}\frac{z}{R}.$ This phase accumulation may result in an interference between the different LG modes, for example, $\left( {\psi _{ - \ell } + \psi _\ell } \right) \propto \cos [|\ell |\phi + \varphi (|\ell |,z)]$.

### Anisotropic correlation length of magnetic fluctuations

While the control of twisted magnons via external electromagnetic fields is crucial for applications, one may wonder about the stability of the FM state to twisted excitations. To explore this aspect we have to study the influence of vorticity on the magnetic fluctuations and in particular the correlation length. Both are indicators on how magnetic ordering reacts to excitations^46^. Let us start with the Ginzburg–Landau free energy density of the FM waveguide at temperature *T* (in unit of 1/M^2^), ${\cal{F}} = \frac{{Ja^2}}{2}({\bf{\nabla }}{\mathbf{m}})^2 + (k_{\mathrm{B}}T - \frac{{K_{\mathrm{s}}}}{2})m_{z}^{\mathrm{2}} + K_4m^4 - B_{z}m_{z}$ (with *k*_B_ is Boltzmann constant and |*m*_*z*_| ≤ 1). The quartic term with *K*_4_ > 0 stabilizes the long-range FM ordering below *T*_c_ ≈ *K*_s_/2*k*_B_. Because of the uniaxial anisotropy and the cylindrical demagnetizing field, the SU(2) spin symmetry is reduced to SO(2)$\times {\Bbb Z}_2$. Low-energy Goldstone modes are due to the continuous SO(2) symmetry.

For *T* < *T*_c_ the saddle point approximation delivers the mean-field value of *m*_*z*_ in the absence of an external magnetic field: $\bar m_{z}(T) = \pm \sqrt {k_{\mathrm{B}}(T_{\mathrm{c}} - T)/2K_4}$ in the FM sector. The stiffness of deformations around the saddle point solution is inferred from the longitudinal (along $\widehat {\mathbf{e}}_z$) and transverse (along $\widehat {\mathbf{e}}_{r}$ and $\widehat {\mathbf{e}}_\phi$) fluctuations^46^, ${\mathbf{m}} \simeq [{\cal{R}}({\mathbf{r}}),\Phi ({\mathbf{r}}),\bar m_{z} + {\cal{Z}}({\mathbf{r}})]$. The fluctuations energy cost, up to second order, is $\delta {\cal{F}} = \;\frac{{Ja^2}}{2}\left[ {({\bf{\nabla }}{\cal{R}})^2 + ({\bf{\nabla }}\Phi )^2 + ({\bf{\nabla }}{\cal{Z}})^2} \right] - 2k_{\mathrm{B}}(T - T_{\mathrm{c}}){\cal{Z}}^2.$ For non-diffracting Bessel modes at $T \ll T_{\mathrm{c}}$, we find the anisotropic characteristic length scales, $\frac{1}{{\xi _{z}}} \simeq \sqrt {\frac{{2K_{\mathrm{s}}}}{{Ja^2}}} ,\frac{1}{{\xi _{\mathrm{r}}^{\ell n}}} \approx \frac{{\chi _{\ell {n}}}}{R}\,\,{\mathrm{and}}\,\,\frac{1}{{\xi _\phi ^\ell }} \approx \frac{\ell }{R}$, where $\chi _{\ell {n}}$ is the *n*th root of the Bessel prime function ${\mathrm{d}}J_\ell (r)/{\mathrm{d}}r|_{{r} = {R}} = 0$. The definition of *χ*_00_ = 0 gives $\frac{1}{{\xi _{\mathrm{r}}^{00}}} = 0$ and $\frac{1}{{\xi _\phi ^0}} = 0$, which implies that the transverse excitations correspond to the Goldstone modes of the SO(2) symmetry.

For the paramagnetic region *T* > *T*_c_, the quartic *K*_4_ term is not relevant and the mean-field equilibrium solution is $\bar m_{z} = B_{z}/k_{\mathrm{B}}(T - T_{\mathrm{c}})$. For transverse fluctuations around the saddle point solution $\bar m_{z}$, we write ${\mathbf{m}} \simeq \sqrt {1 - \rho ^2} \widehat {\mathbf{e}}_{z} + \rho \widehat {\mathbf{e}}_{r},$ with 0 ≤ *ρ* ≤ 1. Substituting in $\delta {\cal{F}}$ and neglecting constants and higher order terms in *ρ* leads to $\delta {\cal{F}}_\rho = \frac{{Ja^2}}{2}({\bf{\nabla }}\rho )^2 - \alpha (T)\rho ^2 + \beta \rho ^4$ with   *α*(*T*) = [*k*_B_*T* − (*K*_s_ + *B*_*z*_)/2] and *β* = *B*_*z*_/8. Interestingly, transverse fluctuations are most probable not at the boundary *ρ* = 0 but at $\rho _0 = \sqrt {\alpha /2\beta }$, the global minimum of the free energy $\delta {\cal{F}}_\rho$. For cylindrical waveguide the transverse fluctuations are subject to a non-zero restoring force and the correlation length is $\frac{1}{{\xi _{r}}} \simeq \sqrt {\frac{{4\alpha (T)}}{{Ja^2}}} .$ Thus, the transverse scattering density is Lorentzian.

### Generation of twisted magnon beams

In Fig. 2 we demonstrated how to trigger twisted magnons via magnetic fields. In fact, there are various other ways to accomplish this goal: one may use, for instance, an engineered magnetic spiral phase plate or fabricate a hologram such as a pitch fork for scattering of incoming magnons before entering the waveguide. Another possibility is the following (cf. also Fig. 1): let us consider a uniformly charged thin wire with a linear charge density *λ*_e_ located in the region *z* ∈ [*d*_1_, *d*_2_] on the axis of a FM cylindrical waveguide. The radial electric field ${\cal{E}}({\mathbf{r}}) = \frac{{\lambda _{\mathrm{e}}}}{{2\pi \epsilon r}}\widehat {{e}}_{r}$ in the FM nanowire within *z* ∈ [*d*_1_, *d*_2_] ($\epsilon$ is the electric permittivity of the FM). For *z*-polarized magnons, the interaction of magnons with the radial electric field ${\cal{E}}$ involves the vector potential ${\mathbf{A}}_{\mathrm{e}}({\mathbf{r}}) = - {{\cal{E}}} \times \widehat {\mathbf{e}}_{{z}}/c = \frac{{\lambda _{\mathrm{e}}}}{{2\pi \epsilon cr}}\widehat {\mathbf{e}}_\phi ,$ which is intimately related to the so-called Dirac phase for magnons moving along a contour *C*, $\Phi _{\mathrm{D}} = - \frac{{q_{\mathrm{m}}}}{\hbar }{\int}_{C} {{\mathbf{A}}_{\mathrm{e}}} ({\mathbf{r}})\cdot {\mathrm{d}}{\mathbf{r}},$ producing a phase structure $\exp ({\mathrm{i}}\ell \phi )$ with the quantized number $\ell = - \frac{{q_{\mathrm{m}}\lambda _{\mathrm{e}}}}{{2\pi \hbar \epsilon c}}$. Similar to twisted electron beams^47^, the twisted magnons can be produced by considering a transition of a conventional magnon with ($\ell = 0$) in the region *z* ≤ *d*_1_ without any flux (*λ*_e_ = 0) to the region *z* ∈ [*d*_1_, *d*_2_] with the non-zero flux (*λ*_e_ ≠ 0). For an estimate, we have $\frac{{q_{\mathrm{m}}\lambda _{\mathrm{e}}}}{{2\pi \hbar \epsilon c}}\sim 5.6\lambda _{\mathrm{e}} \times 10^{ - 15}$ with the linear charge density *λ*_e_ in unit of e/m. For ~10^22^ cm^−3^ carrier density in copper and magnon wavelength (governed by the exchange interaction) ~μm, the linear density *λ*_e_ ~ 10^14^–10^16^ e/m is experimentally feasible.

### Demonstration of OAM magnon beams evolution

**Fig. 4:**
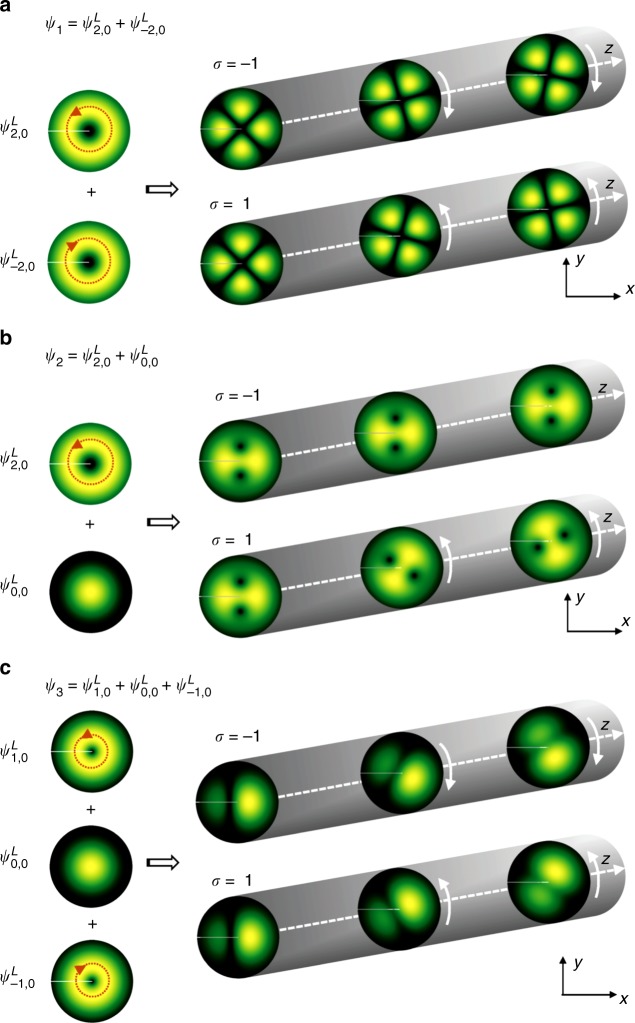
Propagation of Laguerre–Gaussian (LG) eigenmodes along a magnonic waveguide. **a** orbital angular momentum (OAM)-balanced, **b** OAM-unbalanced, and **c** mixed LG eigenmodes. *σ* denotes the direction of the applied electric field

As evidenced, twisted magnon beams are controlled in numerous ways including external electric and magnetic fields, geometry design and holographic means. For instance, with suitably designed holograms, different superpositions of twisted beams can be created such as the OAM-balanced superposition. This is determined by two LG eigenmodes with the same radial number *n* but opposite topological charges $\pm \ell$, meaning $\psi _1 = \psi _{ - \ell ,{n}}^L + \psi _{\ell ,{n}}^L.$ This superposition has zero net canonical OAM 〈*L*_*z*_〉 = 0 exhibiting a flower-like symmetry pattern: $|\psi _1|^2 \propto \cos ^2(\ell \phi )$ at *z* = 0, as shown in Fig. 4a. The OAM-unbalanced superposition consists of two LG eigenmodes with the same radial number *n* but different topological charge 0 and $\ell$ means that $\psi _2 = \psi _{0,{n}}^L + \psi _{\ell ,{n}}^L.$ This superposition carries a finite net canonical OAM, 〈*L*_*z*_〉 ∝ *l*, and is characterized by a pattern with $\ell$ off-axis vortices (cf. Fig. 4b).

In the paraxial approximation with a relatively small transverse kinetic energy and considering that $\Omega _{\mathrm{e}} \propto \sigma |{\cal{B}}|$, we infer the allowed wave number as *k*_*z*_ ≃ *k* + Δ*k*_*z*_, with $\Delta k_{\mathrm{z}} = - [\sigma \ell + (2n + |\ell | + 1)]/z_{\mathrm{e}}$. Here $z_{\mathrm{e}} = w_{\mathrm{e}}\sqrt {E/\hbar |\Omega _{\mathrm{e}}|}$ is a characteristic longitudinal scale. On the propagation distance *z*, the electric field modifies the longitudinal wave vector resulting in an additional phase *φ* = Δ*k*_*z*_*z*, which strongly depends on the direction (*σ*) of the applied electric field. As for $\ell \sigma > 0$, the induced phase *φ* shows a linear dependence on the topological charge $\ell$. In contrast, for $\ell \sigma < 0$, *φ* = −(2*n* + 1)*z*/*z*_e_ is independent of $\ell$. These phenomena are caused by partial cancellation of counter-circulating azimuthal currents produced by the beam with ${\mathrm{exp}}(i\ell \phi )$ and by the twist vector potential **A**_e_.

The propagation of twisted magnon beams in the waveguide can give rise to a Faraday effect similar to the case of electron vortex states^32,33^. As for the OAM-balanced superpositions, the induced phases for the modes $\psi _{ \pm \ell ,{n}}^L$ reads $\varphi _ \pm = \mp \sigma \ell z/z_{\mathrm{e}}$, which results in rotation of the interference pattern by the angle Δ*φ* = *σz*/*z*_e_, as displayed in Fig. 4a. For the OAM-unbalanced superpositions, the mode $\psi _{\ell ,{n}}^L$ acquires an additional phase $\Delta \varphi = - (\sigma \ell + |\ell |)z/z_{\mathrm{e}}$, as compared to the $\psi _{0,{n}}^{L}$ mode. The superposition with $\sigma \ell > 0$ shows a rotation of the interference pattern by the angle Δ*φ* = 2*σz*/*z*_e_, as demonstrated by the lower panels in Fig. 4b with *σ* = −1, whereas the superposition with $\sigma \ell < 0$, has no rotation at all (Δ*φ* = 0, as evidenced by the *σ* = −1 panels in Fig. 4b.

All these electric field tunable phases, including the mixed cases with $\psi _3 = \psi _{ - \ell ,{n}}^L + \psi _{0,{n}}^L + \psi _{\ell ,{n}}^L$, as shown in Fig. 4c, indicate rich magneto-electric patterns of the interference intensity, which also may serve as a marker for identifying the twisted magnon beam.

## Discussion

Imprinting an orbital angular momentum on a magnon beam puts a new twist on magnonics, as OAM can be functionalized as an independent robust, meaning damping resistant parameter. Together with the fact that OAM can be tuned to very large values, the twisted magnon beams offer new opportunities for reliable multiplex communications. The susceptibilities of the OAM-carrying magnons to electric and magnetic fields are thereby a key advantage, as they offer versatile tools to extract and steer the flow of information. Further immediate consequences of the topological nature of the discovered twisted beams are that the internal global phases, related to OAM, result in a controllable interference pattern when such beams interfere. This we may employ to imprint spatially modulated magnon density on a length scale well below the beam’s extensions. The parameters of the twisted beams are directly reflected in the magnon density spatio-temporal pattern. Thus, in addition to the well-established, energy-saving applications of magnonics and magnon-based spintronics, this new offspring is useful for OAM-based, electrically controlled functionalities in magnonic-based information transfer and processing. Future studies are focused on the scattering of twisted magnon beams from a magnetization vortex core^48^ and its possible application in a long distance (at short wavelength^49^) electrically controlled multiplex communication channel between different vortex cores.

## Methods

### Micromagnetic simulations

We used the open-source, graphical processing unit (GPU)-accelerated software package MuMax^3^^50^ for the micromagnetic simulations. The (0.4 × 0.4 × 2.0) μm^3^ large system is discretized into (100 × 100 × 500) cubic cells with a size of (4 nm)^3^. The spatial profile of the excitation field applied at the *z* = 0 layer reads $B_\ell (\rho ,\phi ,t) = \Re \left\{ {\hat eB_{{\mathrm{max}}}(\frac{\rho }{{w_0}})^{|\ell |}\exp \left[ {\frac{{|\ell |}}{2}\left( {1 - \frac{{\rho ^2}}{{w_0^2}}} \right)} \right]\exp \left[ { - i(t\omega _{B} + \ell \phi )} \right]} \right\}$. The waist parameter *w*_0_ denotes the distance of maximum field strength *B*_max_, $\hat e$ corresponds to the polarization vector, which is equal to $\hat e_{\mathrm{x}}$ in our case, whereas *w*_0_ = 75 nm. The time evolution is computed using a Dormand–Prince algorithm with adaptive time-steps. To avoid artifacts due to finite size simulations, we apply open boundary conditions (disregard demagnetizing fields).

## Supplementary information

Peer Review

Description of Additional Supplementary Files

Supplementary Movie 1

## Data Availability

Figures 1, 3, and 4 have been produced from the equations given in text. Derived data that support the findings of this study are available from the corresponding author upon request.

## Code availability

For obtaining the results plotted in Fig. 2 as well as the movie file, we used the open-source, GPU-accelerated software package MuMax^3^^50^ for the micromagnetic simulations. For reproducing the data, one has to use the paramter for YIG, as given in the text and discretize the (0.4 × 0.4 × 2.0) μm^3^ large cylinder into (100 × 100 × 500) cubic cells with a size of (4 nm)^3^. Full details on the code are in ref ^50^.
