# Supplementary material for: Twisted magnon beams carrying orbital angular momentum
Source: Nat Commun. 2019 May 7;10:2077. doi: 10.1038/s41467-019-10008-3 (PMC6504950; doi:10.1038/s41467-019-10008-3)
Supplement: Supplementary file 2 — Description of Additional Supplementary Files [file 41467_2019_10008_MOESM2_ESM.docx]

**Description of Additional Supplementary Files**

File Name: Supplementary Movie 1

Description: Twisted magnon dynamics in a 2 micrometer long cylindrical waveguide of the insulting magnet yttrium iron garnet that has a diameter of 0.4 micrometer. The video shows the x component of the triggered magnetization mx at the middle of the waveguide. The twisted magnon beam is launched by acting on one end with radio-frequency magnetic field with a peak amplitude of 10 mT and a frequency of 5 GHz.
