# Supplementary material for: Twisted magnon beams carrying orbital angular momentum
Source: Nat Commun. 2019 May 7;10:2077. doi: 10.1038/s41467-019-10008-3 (PMC6504950; doi:10.1038/s41467-019-10008-3)
Supplement: Supplementary file 1 — Peer Review [file 41467_2019_10008_MOESM1_ESM.pdf]

Reviewers' comments:

Reviewer #1 (Remarks to the Author):

Review of the article entitled
"Twisted magnon beams carrying orbital angular momentum"

In this article, authors discuss the behavior of magnons inside a cylindrical ferromagnetic guide which is used to create orbital angular momentum carried by magnons. Starting from the basic description of magnons in a ferromagnetic media, the authors describe well known behavior of magnons. By using a quantum mechanics tool and using the symmetry of a cylinder, they end to the ground new result of this article: due to the symmetry of the system magnons exist with a specific shape of non-diffracting Bessel modes quantized with l the order of Bessel function describing the twisted magnon beams. In analogy to optical twisted beams, authors show that this l number corresponds to an orbital angular momentum carried by the twisted magnon beams. From this point, authors discuss the influence of damping (see comments below), of an electric field applied to the ferromagnetic cylinder applied along the cylinder axis. From the coupling between twisted magnonic beams and electrical field, they discuss the behavior of the twisted magnonic beam under such an electric field with the displacement of the beams in analogy with Hall effect for electrons (authors introduced the naming of magnon Hall effect), the generation of twisted magnon beams, and the axial evolution of the shape of linear combinations of twisted magnon beams with different orbital angular momentum.

I would ask authors minor changes:
Add a new figure/Modify figure 1 in order to make it easy to understand what are the sample geometry and the ferromagnetic ground state assumed in order to derive the existence of twisted magnon beams.
Clarify the discussion on the influence of the damping which is said to be non affecting the OAM, although the damping term is shown to be proportional to a function ρ_l(r) introduced in Eq 6 but not explicited.
Specify in the discussion how broad their results are: specify the insulating character of the material
In-line with the former point, give some basic numerical applications using real material parameters. This would be welcomed in the discussion to give insights on how experiments should be performed in order to measure/evidence these twisted magnon beams.
Check the typos in the text
Avoid expressions such as "clearly", "well-known", etc…

As a concluding remark, this interesting theoretical approach of magnons in cylinder made of ferromagnetic insulator described in this article deserves publication in Nature Communications after small changes according to the points raised above.

Reviewer #2 (Remarks to the Author):

The manuscript by Jia et al. presents a theoretical proposal for generating twisted magnon beam exploiting the Aharonov-Casher effect. The main finding is that starting from fundamental equations for spin dynamics it is shown how to generate and quantify orbital angular momentum beams in magnonic wave guides. Moreover a study on the magnonic Hall effect as well as on the anisotropic correlation length of the magnetic fluctuations is brought to light.

This manuscript offers interesting results to the spintronic community, especially in view of potential experimental applications. The claims are novel and convincing but the authors have to appropriately discuss them in the context of previous literature. However the manuscript in its present form seems more addressed to a specialised audience and in my opinion it also could be shortened to aid

communication of the most important findings.

Therefore, I believe that this manuscript, upon revision as appropriate that aims at reach also non specialised readers, is suitable for the journal of Nature Communications. Upon resubmission, the authors are suggested to carefully consider the following comments:

1) The introduction needs to be rewritten in order to present in the context of the previous and present literature the current interest toward the topic illustrated.
2) The sections about the magnon Hall effect and the anisotropic correlation function seem to be unbound from the rest of the manuscript and too specific for a broad audience. I suggest to put the two questions in a general framework in which became clear the utility and the interest of such results highlighting the main findings. For example, which is the difference between the OAM dependent Hall effect and the usual magnon Hall effect?
3) Regarding the text, I also believe that it is more appropriate for the Nature Communications journal to include a final section that is more a discussion than simply a summary with a list of the major obtained results.
4) I think that the proposal is very interesting but I think that the manuscript would need an adjoint value that improve the quality or novelty for the Nature Communications journal. It could be beneficial for example to provide a detailed discussion on the link between theory and experiment in order to have a proposal that could be directly exported to a laboratory.

Some technical comments in order to enlarge the audience also to non specialised readers:
5) Under which experimental conditions the hypothesis done in the beginning that longitudinal excitations are much higher in energy than transversal one is verified and reasonable? And why?
6) Looking at Fig. 2 and Fig. 3, what would change if different n and l are considered? Moreover I think that Fig. 2 could be improved adding a legend for the color map or using a different way to describe the different situations represented in Fig. 2a e 2b, that in my opinion is not so clear for a non specialised reader.
7) The temperature appears for the first time in the section devoted to the anisotropy. Which is the role of the temperature in all the results previously presented?

Reviewer #3 (Remarks to the Author):

In their manuscript entitled "Twisted magnon beams carrying orbital angular momentum" Chenglong Jia et al report on the propagating spin waves that carry a definite and electrically tunable orbital angular momentum (OAM). The external tuning of topological charge associated with OAM of a particular beam was shown.

I think the manuscript is clearly structured and the analytical data are discussed in detail. That is why I support the publication in Nature Communication
after some improvements of the manuscript.
Moreover, the manuscript should be slightly revised to improve its readability. I also suggest that the authors consider the following issues:

1) The authors describe the basic physics and types of orbital angular maomentum and it will be more instructive if the analogy with optical angular momentum will present in the Introduction to do it more accessible for general audience.

2) The first part of the introduction is devoted to
the magnon transport and versatile magnonic
logic circuits and their functionalities. Since the author cite the Ref[1] and Ref[3], it will be more instructive if [Phys. Rev. Lett. 120, 257203 (2018)]
is also contained in the reference list along with Refs.1-5.

3) The authors cite Ref.3 [Wang, Q. et al Reconfigurable nanoscale spin-wave directional coupler, Sci. Adv. 4, e1701517 (2018)] in the Introduction, however in the context of the devices for planar spin-wave transport by the means of coupled spin waves. To be honest, I suggest to also include Ref [Applied Physics Letters 107, 202405 (2015)] and Ref[Phys. Rev. B.,96, 144428 (2017)] from group of Prof Nikitov, which were the first who shows the possibility of realization of spin-wave directional coupler.

4) The theory was constructed for the case of a given linear and constant charge density which determines all effects studied in the system. Will occur
the redistribution of the charge density in the process of the movement of a twisted magnon along the line
charge? If yes, then what effects it can lead to.

5) The considered system consists of the tube-waveguide ("Noting that our twisted beam is localized within the wave guide..."). It is not clear from the manuscript how the for of the waveguide will affect the twisted beam. If the authors use the definition of the Poynting vector, it is also worth to mention the boundary condition for the equation and system under consideration. The comments about shape anisotropy should also be added.

I hope these corrections will improve the manuscript.

<u>Response to the reviewers' reports:</u>

To all reviewers:

[**Micromagnetic simulations**]

To establish a link to experiment, reviewer 1 and reviewer 2 suggested running numerical simulations for a realistic experimental setup with material-specific parameters. We conducted, tested and analyzed this case and included the main findings in the revised version. In short, we have performed micromagnetic simulations for a cylindrical waveguide made of the insulating magnet yttrium iron garnet (YIG). The waveguide has a 0.4 μm diameter and is 2.0 μm in length. The material parameters for YIG are: the saturation magnetization is $M_s = 1.4 \times 10^5$ A m^{-1}, the exchange stiffness constant is $A_{ex} = 3 \times 10^{-12}$ J m^{-1}, and the Gilbert damping $\alpha = 0.01$. The ground state equilibrium magnetization is oriented along the $+z$ axis. The system is then excited locally at one end with a twisted magnetic B-field, linearly polarized in the x-direction with a field amplitude $B_{max} = 10$ mT and a field frequency $\omega_B = 5$ GHz. Figure r1(a) shows a snapshot of the x-component of the magnonic excitations propagating after 2 ns. The details are illustrated by slices along the waveguide in Figure r1(b). The media files enclosed to the submission evidence nicely how the OAM-carrying magnonic wave spirals along the wave guide axis. As follows from the general analysis, we find that damping causes a magnon density decay along the YIG waveguide. In contrast, the associated OAM, ℓ as evaluated from the pseudo Poynting vector is indeed independent of the magnon density and is conserved during the spin wave propagating (cf. Figure r1 c).

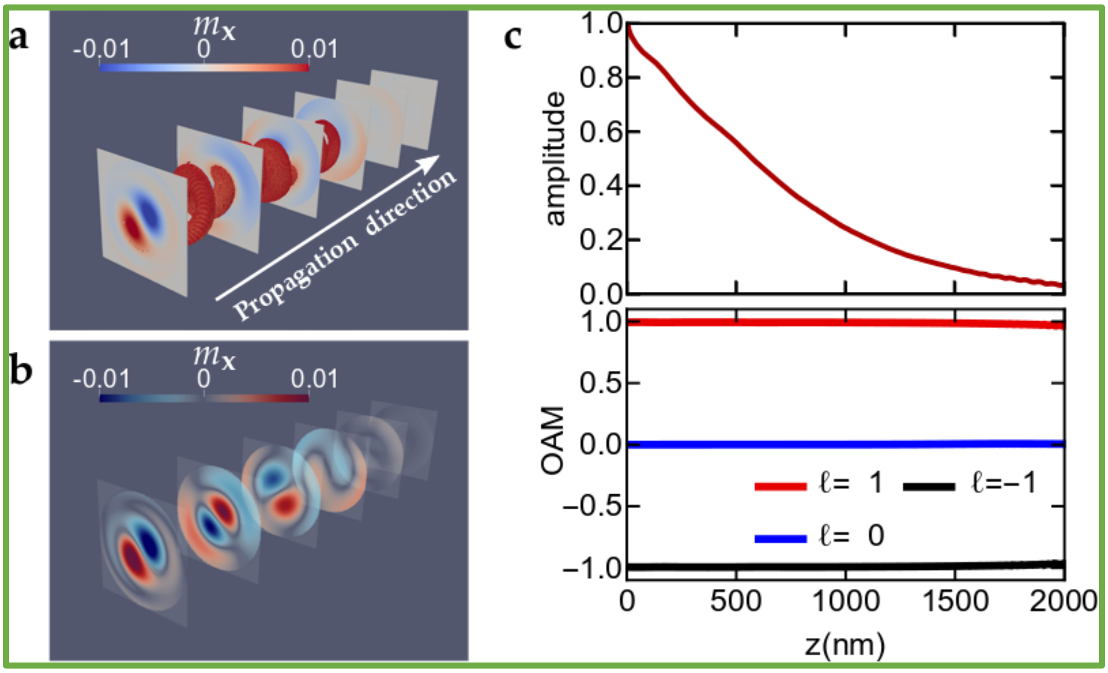

Figure r1. Spin wave propagating along a YIG cylindrical waveguide. **a)** Snapshot of the magnon beam after 2 ns for the x-component of the magnetization excitations. **b)** Spatial distribution of the excitation modes along the waveguide. **c)** The z-resolved amplitude and orbital angular momentum of spin waves. In **a**, **b**, and **c**, the spin wave is excited locally at one end by a twisted rf magnetic field with the amplitude $B_{max} = 10$ mT and the frequency $\omega_B = 5$ GHz (please see the main text for more details). For the complete time evolution of m_x at the transversal slice $z = 1\mu m$, pelase see the Supplementary Videos.

Reviewer #1 (Remarks to the Author):

[Remarks 1-0] In this article, authors discuss the behavior of magnons inside a cylindrical ferromagnetic guide which is used to create orbital angular momentum carried by magnons. Starting from the basic description of magnons in a ferromagnetic media, the authors describe well known behavior of magnons. By using a quantum mechanics tool and using the symmetry of a cylinder, they end to the ground new result of this article: due to the symmetry of the system magnons exist with a specific shape of non-diffracting Bessel modes quantized with l the order of Bessel function describing the twisted magnon beams. In analogy to optical twisted beams, authors show that this l number corresponds to an orbital angular momentum carried by the twisted magnon beams. From this point, authors discuss the influence of damping (see comments below), of an electric field applied to the ferromagnetic cylinder applied along the cylinder axis. From the coupling between twisted magnonic beams and electrical field, they discuss the behavior of the twisted magnonic beam under such an electric field with the displacement of the beams in analogy with Hall effect for electrons (authors introduced the naming of magnon Hall effect), the generation of twisted magnon beams, and the axial evolution of the shape of linear combinations of twisted magnon beams with different orbital angular momentum. I would ask authors minor changes.

[Remarks 1-1] Add a new figure/Modify figure 1 in order to make it easy to understand what are the sample geometry and the ferromagnetic ground state assumed in order to derive the existence of twisted magnon beams.

[Reply 1-1] Figure 1 is updated accordingly. The new Fig.2 and the supplemental videos offer also an additional possibility for visualizing the setup and how twisted magnons emerge and propagate.

[Remarks 1-2] Clarify the discussion on the influence of the damping which is said to be non-affecting the OAM, although the damping term is shown to be proportional to a function ϱ_l(r) introduced in Eq 6 but not explicited.

[Reply 1-2] The damping term $\alpha\mathbf{m} \times d\mathbf{m}/dt$ can be accounted for in the (magnonic) Schrödinger equation (Eq.3) by the substitution $\partial_t \to (1 + i\alpha)\partial_t$, which results in a time-decaying magnon density $\rho_\ell(r,t) = \rho_\ell(r,t_0)exp[-2\alpha\omega(t - t_0)]$. The OAM, ℓ is however, given by $\langle\mathbf{L}\rangle = \langle\mathbf{r} \times \boldsymbol{P}\rangle_S = \hat{e}_z \frac{\int \ell\rho_\ell(r,t)dS}{\int \rho_\ell(r,t)dS} = \ell\hat{e}_z$ where the integral runs over the cross section S of the waveguide. As we see, ℓ is well-defined and is conserved during the precessional damping of magnonic density. This argument is supported by the full-numerical simulations in Fig.1r. Both, the above arguments and the numerical results are included in the revised version.

[Remarks 1-3] Specify in the discussion how broad their results are: specify the insulating character of the material.

[Reply 1-3] With the new additions to the manuscript on the various ways to trigger twisted magnon beams and also the full numerical results giving further credibility to the general model, we think twisted magnon beams have a similar general character as twisted photon beams. They can be excited in ferromagnetic insulating and ferromagnetic metallic wave guides (similar results we achieved for a metallic waveguide made of FeB). The magnetoelectric effects discussed in the manuscript via the Aharanov-Casher effect concern mostly the insulating wire. However, as pointed out in the revised version for the metallic case, we still have a nontrivial magnetoelectric effect driven by a step electric potential at the surface due to the electrostatic screening.

[Remarks 1-4] In-line with the former point, give some basic numerical applications using real material parameters. This would be welcomed in the

discussion to give insights on how experiments should be performed in order to measure/evidence these twisted magnon beams.

[Reply 1-4] Thank you for this suggestions, we conducted the requested micromagnetic simulation for a YIG-waveguide as described above and included the findings in the revised version. Another micromagnetic simulations performed for a *metallic* FeB waveguide evidence very similar twisted magnon beams, as those demonstrated in Fig.r1 for *insulating* YIG.

[Remarks 1-5] Check the typos in the text. Avoid expressions such as "clearly", "well-known", etc…

[Reply 1-5] We agree to these remarks and recommendations.

[Remarks 1-6] As a concluding remark, this interesting theoretical approach of magnons in cylinder made of ferromagnetic insulator described in this article deserves publication in Nature Communications after small changes according to the points raised above.

[Reply 1-6] Once again, we thank you very much for your comments and suggestions that guided us in improving the paper.

Reviewer #2 (Remarks to the Author):

[Remarks 2-0] The manuscript by Jia et al. presents a theoretical proposal for generating twisted magnon beam exploiting the Aharonov-Casher effect. The main finding is that starting from fundamental equations for spin dynamics it is shown how to generate and quantify orbital angular momentum beams in magnonic wave guides. Moreover a study on the magnonic Hall effect as well as on the anisotropic correlation length of the magnetic fluctuations is brought to light.

This manuscript offers interesting results to the spintronic community, especially in view of potential experimental applications. The claims are novel and convincing but the authors have to appropriately discuss them in the context of previous literature. However the manuscript in its present form seems more addressed to a specialised audience and in my opinion it also could be shortened to aid communication of the most important findings.

Therefore, I believe that this manuscript, upon revision as appropriate that aims at reach also non specialised readers, is suitable for the journal of Nature Communications. Upon resubmission, the authors are suggested to carefully consider the following comments:

[Remarks 2-1] The introduction needs to be rewritten in order to present in the context of the previous and present literature the current interest toward the topic illustrated.

[Reply 2-1] Thank you for this important hint. The introduction part is rewritten as suggested.

[Remarks 2-2] The sections about the magnon Hall effect and the anisotropic correlation function seem to be unbound from the rest of the manuscript and

too specific for a broad audience. I suggest to put the two questions in a general framework in which became clear the utility and the interest of such results highlighting the main findings. For example, which is the difference between the OAM dependent Hall effect and the usual magnon Hall effect?

[Reply 2-2] Indeed the motivations for these sections were not clearly written: For applications two aspects are crucial, a) can we steer externally and detect the twisted magnons?. The magnon Hall effect offers a possibility to deflect the flow of magnons transversally by external fields and then detect this deflection by standard means for sensing magnon accumulations.

b) How stabile is actually the ferromagnetic order itself to twisted magnetic fluctuations, meaning does the ferromagnetic state as a whole survives these excitations?. This issue is answered by inspecting the correlation length and magnetic fluctuations around the ferromagnetically ordered state. We rewrote these sections in the paper as to highlight the above motivations.

Compared with the usual magnon Hall effect, the OAM-magnon Hall effect can be tuned by increasing ℓ which is unbounded, in principle thus allowing for a large Hall effect, even with small external driving. In addition, as discussed in the paper, the twisted magnon beam is susceptible to an electric field that can be used to control the magnon Hall effect electrically.

[Remarks 2-3] Regarding the text, I also believe that it is more appropriate for the Nature Communications journal to include a final section that is more a discussion than simply a summary with a list of the major obtained results.

[Reply 2-3] We followed the recommendations and added a detailed discussions and an outlook on this topic.

[Remarks 2-4] I think that the proposal is very interesting but I think that the manuscript would need an adjoint value that improve the quality or novelty for the Nature Communications journal. It could be beneficial for example to provide a detailed discussion on the link between theory and experiment in order to have a proposal that could be directly exported to a laboratory.

[Reply 2-4] Following the suggestions, we performed micromagnetic simulations for a YIG-waveguide, routinely used in experiments and demonstrated the basic properties of twisted magnon beams for a cylindrical waveguide. The main findings are presented in Fig.r1 and also included in the revised version.

[Remarks 2-5] Some technical comments in order to enlarge the audience also to non specialised readers:
Under which experimental conditions the hypothesis done in the beginning that longitudinal excitations are much higher in energy than transversal one is verified and reasonable? And why?

[Reply 2-5] Longitudinal excitations implies changes in the expectation value of the magnitude of the magnetic moment. This can be due for example to Stoner excitations (particle-hole pair excitations) in itinerant ferromagnets, in which case an electron is excited from an occupied state of the majority-spin band to an empty state of the minority-spin band. The energy need for such excitations can be on the scale of the exchange energy which is much larger than the anisotropy energy that sets the scale for angular excitations away from the ground state magnetization direction while conserving the magnitude of the magnetization vector. The spin waves or magnons correspond to these collective transverse excitations. In the low-temperature regime, the magnetization dynamics is dominated by magnons and the Stoner excitations are frozen. We added more comments regarding this point in the revised paper.

[Remarks 2-6] Looking at Fig. 2 and Fig. 3, what would change if different n and l are considered? Moreover I think that Fig. 2 could be improved adding a legend for the color map or using a different way to describe the different situations represented in Fig. 2a and 2b, that in my opinion is not so clear for a non specialised reader.

[Reply 2-6] For the OAM-dependent magnon Hall effect demonstrated in Fig.2, the transverse deflection depends only and linearly on the vortex topological charge ℓ, and is independent of n. On propagating the twisted magnon beams in the FM waveguide along a distance z, the electric field modifies the longitudinal wave vector resulting in an addition phase $\varphi_{\ell,n} = -\sigma\ell\frac{z}{z_e} - (2n + |\ell| + 1)\frac{z}{z_e}$ with σ being the direction of the applied electric field. The first term is dependent on the vortex topological charge ℓ and the second term is closely related to the Gouy phase. The interference pattern between the LG eigen-modes, shown in Fig.3, are rich and sensitive to ℓ and n.

[Remarks 2-7] The temperature appears for the first time in the section devoted to the anisotropy. Which is the role of the temperature in all the results previously presented?

[Reply 2-7] The thermal fluctuations of the magnetization are enhanced with increasing the temperature. However, the twisted magnon beam and other associated effects do not change qualitatively. The stability of the ferromagnetic order to twisted magnetic thermal fluctuations is explicitly demonstrated in the section on correlation length and magnetic fluctuations.

We thank you again for your kind recommendations that helped us indeed in producing the improved version of the paper.

Reviewer #3 (Remarks to the Author):

[Remarks 3-0] In their manuscript entitled "Twisted magnon beams carrying orbital angular momentum" Chenglong Jia et al report on the propagating spin waves that carry a definite and electrically tunable orbital angular momentum (OAM). The external tuning of topological charge associated with OAM of a particular beam was shown.

I think the manuscript is clearly structured and the analytical data are discussed in detail. That is why I support the publication in Nature Communication after some improvements of the manuscript.

Moreover, the manuscript should be slightly revised to improve its readability. I also suggest that the authors consider the following issues:

[Remarks 3-1] The authors describe the basic physics and types of orbital angular maomentum and it will be more instructive if the analogy with optical angular momentum will present in the Introduction to do it more accessible for general audience.

[Reply 3-1] The introduction has been re-written starting from the optical case, as the suggested, which indeed makes the connection to our study more evident.

[Remarks 3-2] The first part of the introduction is devoted to the magnon transport and versatile magnonic logic circuits and their functionalities. Since the author cite the Ref[1] and Ref[3], it will be more instructive if [Phys. Rev. Lett. 120, 257203 (2018)] is also contained in the reference list along with Refs.1-5.

The authors cite Ref.3 [Wang, Q. et al Reconfigurable nanoscale spin-wave directional coupler, Sci. Adv. 4, e1701517 (2018)] in the Introduction, however in the context of the devices for planar spin-wave transport by the means of coupled spin waves. To be honest, I suggest to also include Ref [Applied

Physics Letters 107, 202405 (2015)] and Ref[Phys. Rev. B.,96, 144428 (2017)] from group of Prof Nikitov, which were the first who shows the possibility of realization of spin-wave directional coupler.

[Reply 3-2] We studied the relevance of these works and agree that they should be added to the reference list, which is done in the revised manuscript.

[Remarks 3-4] The theory was constructed for the case of a given linear and constant charge density which determines all effects studied in the system. Will occur the redistribution of the charge density in the process of the movement of a twisted magnon along the line charge? If yes, then what effects it can lead to.

[Reply 3-3] The emergent electric field E_μ related to the magnetization dynamics reads $E_\mu = \boldsymbol{m} \cdot (\partial_\mu \boldsymbol{m} \times \partial_t \boldsymbol{m})$. We inferred from the profile of $\boldsymbol{m}$ that E_z is small and has a negligible effect on the linear charge. E_ϕ may give rise to a loop current but not to a redistribution of the charge density.

[Remarks 3-5] The considered system consists of the tube-waveguide ("Noting that our twisted beam is localized within the wave guide..."). It is not clear from the manuscript how the for of the waveguide will affect the twisted beam. If the authors use the definition of the Poynting vector, it is also worth to mention the boundary condition for the equation and system under consideration. The comments about shape anisotropy should also be added.

[Reply 3-3] Unlike twisted light or electrons, the magnons, being deviations from an equilibrium magnetization, can not propagate in vacuum but are localized within the magnetic materials. The cylindrical symmetry of the ferromagnetic waveguide results in non-diffracting modes of the magnonic Schrödinger equation (Eq.3 and Eq.4). For a finite length of the waveguide, as has been discussed in the manuscript, the inhomogeneity in the demagnetization field (or shape anisotropy) can cause Magnus-like effects or interference between different eigen-modes.

As for the boundary condition, there are two edges in general: (1) a rigid edge with $\tilde{m}(R) = 0$ ($J_\ell(R) = 0$), and (2) a free edge with $d\tilde{m}(r)/dr|_{r=R} = 0$ (or $dJ_\ell(r)/dr|_{r=R} = 0$). The eigenfrequencies of the Schrödinger equation depend on the specific boundary conditions. In the present study, a free edge is used.

Running a full-fledge numerical simulations for YIG we find that the above general statements are indeed confirmed.

We sincerely thank all three reviewers for their helpful comments and positive feedback. We hope that the revised version covers convincingly most aspects of this new phenomenon and will generate further experimental and theoretical activities on this fascinating topic.

For convenience, changes are marked blue.

REVIEWERS' COMMENTS:

Reviewer #1 (Remarks to the Author):

From their first version of the article entitled "Twisted magnon beams carrying orbital angular momentum", the authors have made several changes which greatly improved the accessibbility of the latter to a broader audience. The micromagnetic simulations although the damping value used for YIG is very pessimistic (a ~ 10-4 are reported for this material) help to make a connection towards experimental realization. Finally I underline the imporved introductory paragraph and conclusion which both highlight the reported results and allow this article to gain in terms of impact.
The article in its new version deserves publication in Nature Communications

Reviewer #2 (Remarks to the Author):

I thank the authors for having addressed all my questions and remarks and especially for having added the new Section “Experimental realization” that in my opinion may improve significantly the value of the paper. For these reasons, I finally recommend the publication in Nature Communications. I hope that in this new version the paper could be appreciated from a broad audience.

Reviewer #3 (Remarks to the Author):

The authors significantly improved the manuscript. The improved version of the paper should be published in the present form.
